# Estimated prevalence and distribution of causes of immune-mediated hypertrophic pachymeningitis in São Paulo, Brazil

**DOI:** 10.1007/s10067-026-08300-x

**Published:** 2026-07-15

**Authors:** Daniela Ohlweiler Brescovit, Sara Terrim, Graziela Aguiar Santos Faria, Vitor Falcão de Oliveira, Tarso Adoni, Suely Kazue Nagahashi Marie, Luiz Henrique Martins Castro, Guilherme Diogo Silva

**Affiliations:** 1 Faculdade de MedicinaUniversidade de Sao Paulo, Sao Paulo, Brazil; 2 Neurology Department, Faculdade de Medicina, Hospital das Clínicas HCFMUSP, Universidade de Sao Paulo, Sao Paulo, SP 05403-000 Brazil; 3 Neurology Department, Hospital Santa Marcelina, Sao Paulo, Brazil; 4 Infectious Disease Department, ICESP, Faculdade de Medicina, Universidade de Sao Paulo, Sao Paulo, Brazil; 5 LIM 15, Neurology Department, Faculdade de Medicina, Universidade de Sao Paulo, Sao Paulo, Brazil

**Keywords:** Granulomatosis, Hypertrophic pachymeningitis, IgG4-related disease, Neurosarcoidosis

## Abstract

**Background:**

The prevalence of hypertrophic pachymeningitis (HP) remains poorly defined worldwide. In Japan, the estimated prevalence is 0.949 cases per 100,000 inhabitants, with most cases related to ANCA-associated vasculitis or IgG4-related disease. In contrast, epidemiological data from other regions, including Latin America, are scarce.

**Methods:**

This study was conducted at a tertiary referral center in the city of São Paulo, Brazil. The prevalence of HP was estimated by comparison with the known prevalence of multiple sclerosis (MS), based on the number of patients with each condition followed at our institution. The etiological distribution of HP was also analyzed. In addition, HP prevalence was compared with that of tuberculous meningoencephalitis, a compulsory notifiable disease and a well-established cause of chronic meningitis in Brazil, using data from the national surveillance system (SINAN).

**Results:**

We identified 55 patients with HP: 36 (65%) idiopathic, 10 (18%) IgG4-related disease, 4 (7%) ANCA-associated pachymeningitis, 4 (7%) probable or definite neurosarcoidosis, and 1 (2%) secondary to rheumatoid arthritis. Among 968 registered MS patients and assuming an MS prevalence of 15 per 100,000 inhabitants in São Paulo, the estimated HP prevalence was 0.85 per 100,000 inhabitants. This estimate was comparable to the prevalence of tuberculous meningoencephalitis in the same region.

**Conclusion:**

The estimated prevalence of HP in São Paulo (0.85 per 100,000) is consistent with reports from other regions and is comparable to that of tuberculous meningeal disease, although with a distinct etiological profile. IgG4-related disease was the most frequent systemic association, differing from previous reports. ANCA-associated HP appears to be less frequent than in other geographic regions, possibly reflecting differences in disease phenotype.

**Table:** 

**Key Points** • *The estimated prevalence of HP in São Paulo was 0.85 per 100,000 inhabitants*. • *IgG4-related disease was the most common systemic disease identified among patients with HP*. • *ANCA-associated HP was less common than reported in Asian and European cohorts*. • *The prevalence of HP was comparable to that of tuberculous meningoencephalitis in São Paulo*.

## Introduction

Hypertrophic pachymeningitis (HP) is a rare inflammatory disorder characterized by inflammatory thickening of the dura mater [1]. Dural inflammation may lead to neurological symptoms primarily related to cranial nerve compression, including headache, facial palsy, diplopia, sensory disturbances, sensorineural hearing loss, and visual impairment [1–3]. Importantly, HP may represent the initial manifestation of an underlying systemic disease, particularly immune-mediated conditions such as IgG4-related disease, sarcoidosis, or vasculitis [1, 4].

A nationwide population-based study conducted in Japan estimated a HP prevalence of 0.949 cases per 100,000 inhabitants, challenging the long-held view of HP as an exceedingly rare condition [2]. In that study, ANCA-associated vasculitis and IgG4-related disease were the most frequent systemic associations. Nevertheless, data regarding HP prevalence and etiological patterns in other regions of the world, including Latin America, remain limited.

The aim of this study was to estimate the prevalence and describe the etiological distribution of immune-mediated HP in São Paulo, Brazil. We hypothesized that HP would not be extremely rare in Brazil, but that the spectrum of associated systemic diseases would differ from that reported in Japan.

## Methods

### Setting

**Fig. 1:**
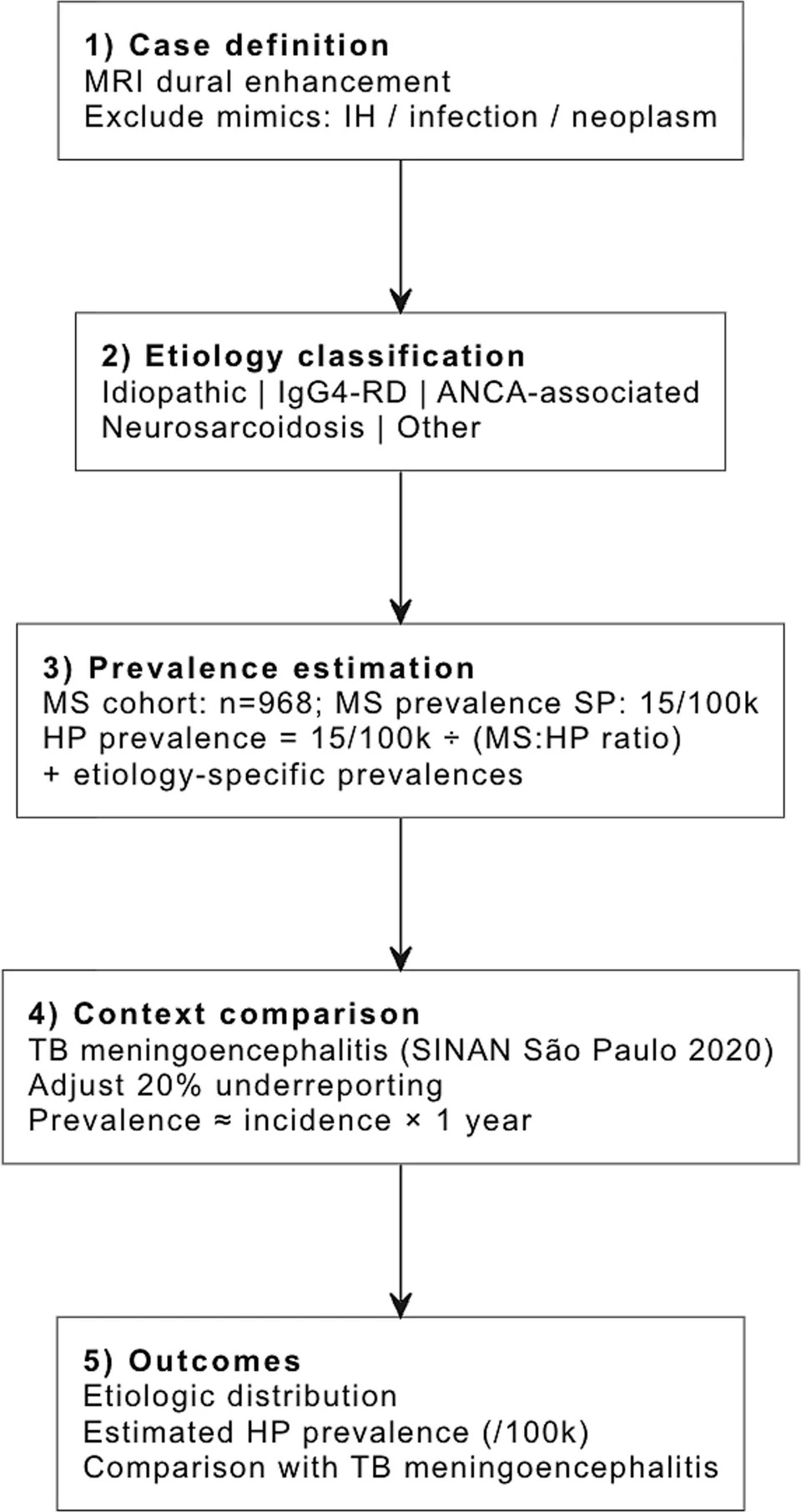
Study design and workflow for case ascertainment, etiologic classification, and prevalence estimation of immune-mediated hypertrophic pachymeningitis in São Paulo, Brazil

This retrospective observational study was conducted at a dedicated outpatient clinic for HP at Hospital das Clínicas, Faculdade de Medicina, Universidade de São Paulo, in 2025 and 2026. The study protocol was approved by the local Ethics Committee (68668723.6.0000.0068). Figure 1 summarizes the study design and workflow.

### Distribution of causes

Medical records of all patients followed at the clinic as of January 19, 2026, were reviewed. Patients were included if they had a diagnosis of HP based on dural enhancement on magnetic resonance imaging, after exclusion of intracranial hypotension, infectious diseases, and neoplastic conditions.

Etiological diagnoses were based on a combination of clinical, imaging, laboratory, and, when available, histopathological findings. All patients underwent autoimmune screening, cerebrospinal fluid analysis, and systemic imaging. Histopathological evaluation was performed whenever a biopsy-accessible site was identified. In our service, routine diagnostic evaluation is performed with chest, abdomen, and pelvis CT scans to identify possible extrameningeal disease and potential biopsy sites, and whole-body FDG-PET scans are performed when the initial tests are negative. Etiological classification incorporated all available follow-up data until the study cutoff date (January 2026).

Patients were classified into five groups: idiopathic, IgG4-related disease, neurosarcoidosis, ANCA-related pachymeningitis, and other identified causes. IgG4-related disease was defined according to the 2019 ACR/EULAR classification criteria [5], with all cases fulfilling the required threshold (≥ 20 points). Neurosarcoidosis was classified according to the 2017 Neurosarcoidosis Consortium criteria, and only patients meeting criteria for definite or probable disease were included [6]. ANCA-associated HP was defined by ANCA positivity or the presence of an ANCA-associated disease, including granulomatosis with polyangiitis, microscopic polyangiitis, or eosinophilic granulomatosis with polyangiitis [7–9]. Patients were classified as idiopathic only after exclusion of infectious, neoplastic, autoimmune, and systemic inflammatory causes following comprehensive evaluation. Patients diagnosed with Tolosa–Hunt syndrome were also classified as idiopathic HP due to the absence of a recognized systemic association.

### Prevalence estimation

Because HP is not a notifiable disease and lacks a single diagnostic test, prevalence estimation is challenging. A pragmatic approach in this context is comparison with a disease of known prevalence using data from a large tertiary referral center [10]. Based on the ratio of patients with each disease treated at the same institution, the prevalence of a rare condition can be estimated.

HP prevalence was estimated by comparison with multiple sclerosis (MS). A population-based study conducted in the city of São Paulo reported an MS prevalence of 15 per 100,000 inhabitants [11], a figure supported by more recent national data [12, 13]. Given that our institution serves as a major referral center for both MS and immune-mediated pachymeningitis in the city, this approach was considered a pragmatic strategy for obtaining an indirect estimate of HP prevalence. Nevertheless, this method assumes broadly comparable referral patterns and case ascertainment between the two conditions, an assumption that may not be fully met given the rarity and diagnostic heterogeneity of HP. To further support this numerical association, we compared our findings with published data from the Federal University of São Paulo (UNIFESP) [14, 15], a second tertiary referral center located in the same city, with a similar level of complexity but a different geographic catchment area than our institution.

To contextualize HP prevalence, we compared it with that of tuberculous meningoencephalitis (a known cause of chronic meningitis in Brazil) [16] using data from the Notifiable Diseases Information System (Sistema de Informação de Agravos de Notificação—SINAN) database, including all confirmed cases reported in the city of São Paulo in 2020 [17]. All confirmed cases of tuberculous meningoencephalitis reported in the municipality of São Paulo in 2020 were included. The same reference year adopted for estimating the prevalence of MS in Brazil was used to ensure temporal alignment between the analyses. Data extraction was performed through the TABNET interface on February 2, 2026. The following filters were applied: state of São Paulo, year of notification (2020), municipality of residence (included only the cases with residence in the city of São Paulo), and confirmed cases. From the available clinical classifications, only cases coded as meningoencephalic tuberculosis were selected. The platform automatically generated a spreadsheet containing the selected variables. To account for underreporting, which has been estimated at approximately 20% for tuberculosis surveillance data in Brazil, the number of reported cases was adjusted by applying a correction factor of 1/0.8, as previously described in national epidemiological studies [18]. The adjusted number of cases was then used to calculate the annual incidence rate by dividing it by the estimated population of the municipality of São Paulo in 2020 and multiplying by 100,000. Because point prevalence data for tuberculous meningoencephalitis are not directly available in surveillance systems, prevalence was estimated using the standard epidemiological relationship between prevalence, incidence, and average disease duration (prevalence = incidence × average disease duration) [19]. An average disease duration of 1 year was assumed, based on national treatment guidelines recommending a 12-month therapeutic regimen for neurotuberculosis in Brazil [20]. Prevalence estimates were expressed as cases per 100,000 inhabitants. In addition, we performed a sensitivity analysis using data from 2017 to 2025 to reduce the potential impact of the COVID-19 pandemic on disease diagnosis and case notification.

## Results

We reviewed the medical records of 65 patients followed at our specialized outpatient clinic for autoimmune hypertrophic pachymeningitis and neurological complications of systemic diseases. Six patients were excluded because they were not under regular follow-up and their diagnostic workup was incomplete. An additional four patients were excluded because they did not demonstrate pachymeningeal enhancement on brain MRI. The final study cohort consisted of 55 patients, whose clinical, radiological, and histopathological findings are summarized in Table 1.

**Table 1:** Demographic, clinical, cerebrospinal fluid, imaging, and histopathological characteristics of patients with hypertrophic pachymeningitis in the study cohort

Characteristic	Total (*n* = 55)	Idiopathic (*n* = 36)	IgG4-RD (*n* = 10)	Sarcoidosis (*n* = 4)	ANCA (*n* = 4)	Rheumatoid arthritis (*n* = 1)
**Demographic features**						
Age at symptom onset, median (IQR), years	45 (35–50)	45.5 (37–49.2)	35 (24–46)	46 (38.5–50)	53 (40.2–57)	64 (64–64)
Female sex, *n* (%)	44 (80)	29 (80)	9 (90)	4 (100)	1 (25)	1 (100)
Time from symptom onset to study inclusion, median (IQR), months	97 (59.5–130)	85 (54.5–115)	116 (64.5–146)	162 (110–217)	122 (96.8–126)	72 (72–72)
**Neurological signs and symptoms**						
Headache, *n* (%)	50 (90)	34 (94)	10 (100)	3 (75)	3 (75)	0
Visual symptoms (II CN), *n* (%)	23 (42)	16 (44)	5 (50)	1 (25)	1 (25)	0
Diplopia or strabismus (III, IV, or VI CN), *n* (%)	32 (58)	23 (64)	6 (60)	0	3 (75)	0
Facial numbness (V CN), *n* (%)	14 (25)	10 (27)	3 (30)	1 (25)	0	0
Facial palsy (VII CN), *n* (%)	11 (20)	5 (14)	4 (40)	1 (25)	1 (25)	0
Hearing loss and/or vertigo (VIII CN), *n* (%)	8 (14)	1 (3)	5 (50)	0	2 (50)	0
Bulbar syndrome (IX–XII CN), *n* (%)	5 (9)	2 (5)	0	2 (50)	1 (25)	0
Focal neurological deficits, *n* (%)	4 (7)	2 (5)	1 (10)	0	0	1 (100)
Seizures, *n* (%)	10 (18)	5 (14)	4 (40)	0	0	1 (100)
Cognitive decline, *n* (%)	2 (3)	1 (3)	1 (10)	0	0	0
**Cerebrospinal fluid findings**						
CSF pleocytosis > 5, *n* (%)	26 (47)	14 (39)	5 (50)	2 (50)	4 (100)	1 (100)
Elevated CSF protein > 40, *n* (%)	27 (49)	14 (39)	5 (50)	3 (75)	4 (100)	1 (100)
CSF cell count, median (IQR), cells/mm^3^	5.5 (2–29.5)	3 (2–18)	6 (3–12)	38 (19–43.5)	180 (136–221)	39 (39–39)
CSF protein, median (IQR), mg/dL	43 (31–70.5)	35 (28.5–56.5)	47 (35–60)	75 (46.5–91.5)	228 (166–326)	31 (31–31)
**MRI findings**						
Cavernous sinus or orbital apex involvement, *n* (%)	34 (62)	25 (69)	6 (60)	3 (75)	0	0
Anterior fossa involvement, *n* (%)	8 (14)	2 (5)	3 (30)	2 (50)	0	1 (100)
Middle fossa involvement, *n* (%)	24 (43)	14 (39)	7 (70)	2 (50)	0	1 (100)
Cerebral convexity involvement, *n* (%)	6 (11)	1 (3)	3 (30)	1 (25)	1 (25)	0
Falx cerebri involvement, *n* (%)	7 (12)	2 (5)	4 (40)	0	1 (25)	0
Tentorial involvement, *n* (%)	13 (23)	5 (14)	4 (40)	2 (50)	2 (50)	0
Posterior fossa involvement, *n* (%)	15 (27)	6 (16)	3 (30)	3 (75)	3 (75)	0
Spinal pachymeningitis, *n* (%)	1 (2)	0	0	0	1 (25)	0
**Histopathological and systemic findings**						
Histopathological confirmation, *n* (%)	30 (54)	12 (33)	10 (100)	4 (100)	4 (100)	0
Meningeal biopsy, *n* (%)	10 (18)	4 (11)	3 (30)	1 (25)	2 (50)	0
Extrameningeal biopsy, *n* (%)	20 (36)	8 (22)	7 (70)	3 (75)	2 (50)	0
Extraneurological/systemic involvement, *n* (%)	20 (36)	7 (19)	7 (70)	3 (75)	2 (50)	1 (100)

Histopathological confirmation was obtained in 30 patients (54%), including 10 patients (18% of the total cohort; 31% of those with histopathological confirmation) who underwent meningeal biopsy. The female-to-male ratio was 4.5:1. The median interval between symptom onset and study inclusion was 97 months (IQR 59.5–130 months). Thirty-six patients (65%) were classified as idiopathic HP, 10 (18%) as IgG4-related disease, 4 (7%) as ANCA-associated pachymeningitis, 4 (7%) as probable or definite neurosarcoidosis, and 1 (2%) as HP secondary to rheumatoid arthritis.

A total of 968 MS patients were included for prevalence estimation. Based on the MS:HP ratio, the estimated HP prevalence was 0.85 per 100,000 inhabitants (95% CI: 0.64–1.11), indicating that MS was approximately 16 times more frequent. As an additional point of reference, data from another tertiary referral center in São Paulo yielded a similar distribution of cases. A study published by the UNIFESP group in June 2025 identified 811 patients with multiple sclerosis [14], whereas a separate study presented in April 2025 reported 51 patients with inflammatory pachymeningitis under follow-up [15]. The resulting multiple sclerosis-to-pachymeningitis ratio was 15.9:1, closely matching the ratio observed at our institution. Although this finding does not eliminate the possibility of referral bias, it provides independent support for the consistency of the relative frequency of both conditions across major referral centers in the city.

Etiology-specific prevalences were estimated at 0.56 per 100,000 for idiopathic HP (95% CI: 0.39–0.77), 0.15 per 100,000 for IgG4-related disease (95% CI: 0.07–0.28), and 0.06 per 100,000 for both neurosarcoidosis and ANCA-associated HP (95% CI: 0.02–0.16). These findings are summarized in Table 2.

**Table 2:** Number of patients by disease and etiology, ratio between diseases, and estimated prevalence of multiple sclerosis and hypertrophic pachymeningitis of different causes in São Paulo, Brazil, with corresponding 95% confidence intervals

Disease		Cases in the referral center	MS/HP ratio	Prevalence (per 100,000)
Multiple sclerosis		968	1	15
Hypertrophic pachymeningitis		55	17.60	0.85 [0.64–1.11]
HP by etiology	Idiopathic	36	26.89	0.56 [0.39–0.77]
	IgG4-related disease	10	96.80	0.15 [0.07–0.28]
	Neurosarcoidosis	4	242.00	0.06 [0.02–0.16]
	ANCA-associated	4	242.00	0.06 [0.02–0.16]
	Rheumatoid arthritis	1	968.00	0.01 [0.00–0.09]

According to SINAN data, 98 cases of tuberculous meningoencephalitis were reported in São Paulo in 2020. Considering the estimated population of 12,325,232 inhabitants for the same year, this corresponds to a crude annual incidence of 0.79 cases per 100,000 inhabitants. After adjustment for an estimated 20% underreporting of tuberculosis cases, the number of cases increased to 123, yielding an adjusted incidence of approximately 1.0 case per 100,000 inhabitants per year. The values observed in 2020 were comparable to those reported in the other years analyzed. The median annual number of cases was 84, suggesting that the pandemic did not lead to substantial underreporting of tuberculous meningoencephalitis. Assuming an average disease duration of 1 year, this incidence estimate corresponds to an approximate prevalence of 1.0 case per 100,000 inhabitants. This value should be interpreted as a contextual estimate derived from incidence data rather than a directly measured prevalence.

## Discussion

In this study, we estimated an HP prevalence of 0.85 per 100,000 inhabitants in São Paulo, Brazil. IgG4-related disease was the most frequent systemic association, followed by neurosarcoidosis and ANCA-associated pachymeningitis.

Our findings corroborate previous evidence that HP is not an exceedingly rare condition. The estimated prevalence closely mirrors that reported in Japan [2], suggesting that overall population prevalence may be relatively similar across regions, despite substantial differences in etiological distribution.

IgG4-related disease accounted for 18% of HP cases in our cohort, a higher proportion than reported in studies from Japan, France, and China [2, 21, 22]. Geographic and ethnic factors may contribute to these differences, although data from Latin America remain limited. The prevalence of HP in IgG4-RD patients is uncertain [23]. As only 2–4% [24] of patients with IgG4-RD have symptomatic HP, the estimated prevalence of IgG4-RD in São Paulo would range from approximately 3.75 to 7.5 cases per 100,000 inhabitants. This range closely matches the reported prevalence of IgG4-RD in the USA (5.3 cases per 100,000 inhabitants) [25].

Neurosarcoidosis was the third most frequent etiology, consistent with the relatively low prevalence of sarcoidosis in Brazil [26]. As only about 1% of patients with sarcoidosis develop HP [27], the estimated prevalence of sarcoidosis in São Paulo would be below 8 cases per 100,000 inhabitants, matching a prior estimate of less than 10 cases per 100,000 inhabitants [26]. In contrast, ANCA-associated HP was less frequent than reported in Asian and European cohorts, possibly reflecting geographic differences in ANCA-associated vasculitis prevalence and serological profiles [22, 28, 29]. In addition, ANCA-associated vasculitis prevalence has been shown to be higher in the northern hemisphere [30].

The prevalence of HP was comparable to that of tuberculous meningoencephalitis, a condition traditionally considered a major cause of chronic meningitis in Brazil [16]. These findings underscore the need to consider immune-mediated HP in the differential diagnosis of chronic meningeal syndromes, even in tuberculosis-endemic regions.

These findings represent the first data on the prevalence of HP in Brazil, and it contributes to a better understanding of its distribution and etiological classification in the country. However, some limitations should be acknowledged. First, this was a single-center study. As a major referral center for both HP and MS in São Paulo, our institution is likely to capture a patient population broadly representative of the city’s referral network. In the Brazilian public healthcare system, referral guidelines recommend that patients with MS be managed in specialized neurology services [31]. Although HP is not specifically addressed in national protocols, it is also considered a high-complexity neurological condition that requires specialized care, including access to magnetic resonance imaging, regular neurological follow-up, and high-cost immunosuppressive therapies. Therefore, both conditions are preferentially referred to tertiary centers such as ours. To further support the representativeness of our findings, we incorporated data from another tertiary center in São Paulo, which reported a comparable proportion of HP and MS cases. However, caution is warranted when extrapolating these results to other regions of Brazil, given potential geographic differences in referral patterns, healthcare access, and disease recognition.

We also assumed that the ratio between MS and HP was similar to that observed at the time of the MS prevalence study. The HP cohort was updated through January 2026, whereas the MS prevalence estimate was derived from an earlier population-based study, supplemented by more recent national data, which suggested that the epidemiological profile of MS has remained relatively stable [12, 13]. However, changes over time in MRI accessibility, diagnostic criteria, disease awareness, referral pathways, and healthcare organization may have influenced case ascertainment for both diseases.

Moreover, we cannot rule out that some idiopathic cases may develop systemic disease during follow-up, although the long median follow-up duration of 97 months reduces the likelihood that systemic manifestations were simply missed. Additionally, IgG4-related disease was defined according to the 2019 ACR/EULAR classification criteria. As a result, some patients with clinical features suggestive of IgG4-related disease who did not meet the classification threshold may have been classified as having idiopathic disease. Finally, as a public referral center for adults, our study did not include patients from the private healthcare system or pediatric population.

It is important to highlight that the prevalence estimate should be interpreted in light of the assumptions underlying our methodology. The comparison with MS was designed to provide an indirect estimate based on a disease with established population prevalence and substantial representation at our institution. However, HP is considerably rarer and more diagnostically heterogeneous than MS, and referral patterns may differ between the two conditions. Consequently, the estimated prevalence reported here should be viewed as an approximation rather than a direct population-based measure. Similarly, the comparison with tuberculous meningoencephalitis was intended primarily for epidemiological context. The estimates derive from different methodological approaches, including referral-center-based prevalence estimates for HP and population-based notification data for tuberculous meningoencephalitis. Furthermore, because direct prevalence data are unavailable for tuberculosis, prevalence was approximated from incidence data assuming a 1-year average disease duration, which may not fully capture the variability in disease course. Therefore, these figures should be interpreted as contextual rather than as evidence of epidemiological equivalence.

We note that the use of distinct epidemiological sources and estimation methods may be viewed both as a limitation and as a reflection of the current reality of chronic meningitis research, where epidemiological data are often derived from heterogeneous methodologies. This heterogeneity reinforces the need for future population-based studies to better characterize the epidemiology of these rare conditions.

## Conclusion

Hypertrophic pachymeningitis appears to be a global disease with similar prevalence across different populations, although its etiological spectrum varies according to regional distribution of systemic diseases. Further multicenter and population-based studies are needed to better define its epidemiology.

## Data Availability

Data used in this manuscript are available upon request.

## References

[1] Hahn LD, Fulbright R, Baehring JM. Hypertrophic pachymeningitis. J Neurol Sci. 15 de agosto de 2016;367:278–83. 10.1016/j.jns.2016.06.024 27423604

[2] Yonekawa T, Murai H, Utsuki S, Matsushita T, Masaki K, Isobe N et al (2014) A nationwide survey of hypertrophic pachymeningitis in Japan. J Neurol Neurosurg Psychiatry julho de 85(7):732–739 10.1136/jnnp-2013-306410 24273222

[3] Brescovit DO, Lucato LT, Castro LHM, Marie SKN, Silva GD (2026) “Eiffel-by-night” sign in hypertrophic pachymeningitis: Clinical and radiological correlates. Clin Neurol Neurosurg março de 262:109291. 10.1016/j.clineuro.2025.109291 41455465

[4] Abrantes FF, de Moraes MPM, RezendeFilho FM, Pedroso JL, Barsottini OGP (2020) A clinical approach to hypertrophic pachymeningitis. Arq Neuropsiquiatr dezembro de 78(12):797–804. 10.1590/0004-282X20200073 33295420

[5] Wallace ZS, Naden RP, Chari S, Choi HK, Della-Torre E, Dicaire JF et al (2020) The 2019 American College of Rheumatology/European League against Rheumatism classification criteria for IgG4-related disease. Ann Rheum Dis janeiro de 79(1):77–87 10.1136/annrheumdis-2019-216561 31796497

[6] Stern BJ, Royal W, Gelfand JM, Clifford DB, Tavee J, Pawate S, et al. Definition and consensus diagnostic criteria for neurosarcoidosis: from the neurosarcoidosis consortium consensus group. JAMA Neurol. 1° de dezembro de 2018;75(12):1546–53. 10.1001/jamaneurol.2018.2295 30167654

[7] Robson JC, Grayson PC, Ponte C, Suppiah R, Craven A, Judge A et al (2022) 2022 American College of Rheumatology/European Alliance of Associations for Rheumatology classification criteria for granulomatosis with polyangiitis. Ann Rheum Dis março de 81(3):315–320. 10.1136/annrheumdis-2021-221795 35110333

[8] Grayson PC, Ponte C, Suppiah R, Robson JC, Craven A, Judge A et al (2022) 2022 American College of Rheumatology/European Alliance of Associations for Rheumatology classification criteria for eosinophilic granulomatosis with polyangiitis. Ann Rheum Dis março de 81(3):309–314. 10.1136/annrheumdis-2021-221794 35110334

[9] Suppiah R, Robson JC, Grayson PC, Ponte C, Craven A, Khalid S et al (2022) 2022 American College of Rheumatology/European Alliance of Associations for Rheumatology classification criteria for microscopic polyangiitis. Arthritis Rheumatol março de 74(3):400–406. 10.1002/art.41983 35106973

[10] Lana-Peixoto MA, Talim NC, Pedrosa D, Macedo JM, Santiago-Amaral J (2021) Prevalence of neuromyelitis optica spectrum disorder in Belo Horizonte, Southeast Brazil. Mult Scler Relat Disord maio de 50:102807. 10.1016/j.msard.2021.102807 33609926

[11] Callegaro D, Goldbaum M, Morais L, Tilbery CP, Moreira MA, Gabbai AA et al (2001) The prevalence of multiple sclerosis in the city of São Paulo, Brazil, 1997. Acta Neurol Scand outubro de 104(4):208–213. 10.1034/j.1600-0404.2001.00372.x 11589649

[12] Moura JA de, Teixeira LA da C, Tanor W, Lacerda ACR, Mezzarane RA. Prevalence of multiple sclerosis in Brazil: an updated systematic review with meta-analysis. Clin Neurol Neurosurg. fevereiro de 2025;249:108741. 10.1016/j.clineuro.2025.108741 39826442

[13] Gonçalves MVM, Siquineli F, Ribas FD, Longo AL, Amaral CH do, Chikota EM, et al. Prevalência de esclerose múltipla em cidades-chave do Brasil. Estudo em Joinville, Sul do Brasil. Arq Neuropsiquiatr. 2021;79:122–6. 10.1590/0004-282X-anp-2020-0101 33759978

[14] Menezes FTL de, Alencar JMD, Lopes AB, Amorim L de S, Portugal RP, Albuquerque FT, et al. The impact of changing diagnostic criteria on disability in a Brazilian multiple sclerosis cohort. Arq Neuropsiquiatr. junho de 2025;83(6):1–9. 10.1055/s-0045-1809662 PMC12196560 40562370

[15] Epidemiological, clinical, and radiological assessment of hypertrophic pachymeningitis in a tertiary neurology center in Brazil (P6–8.017) | Neurology [Internet]. Disponível em: https://www.neurology.org/doi/10.1212/WNL.0000000000211016. Accessed 4 July 2026

[16] Silva GD, Guedes BF, Junqueira IR, Gomes HR, Vidal JE (2022) Diagnostic and therapeutic approach to chronic meningitis in Brazil: a narrative review. Arq Neuropsiquiatr novembro de 80(11):1167–1177. 10.1055/s-0042-1758645 PMC9797267 36577417

[17] TabNet Win32 3.3: TUBERCULOSE - Casos confirmados notificados no Sistema de Informação de Agravos de Notificação - São Paulo [Internet]. Disponível em: http://tabnet.datasus.gov.br/cgi/tabcgi.exe?sinannet/cnv/tubercsp.def. Accessed 3 July 2026

[18] Sousa LMO, Pinheiro RS (2011) Óbitos e internações por tuberculose não notificados no município do Rio de Janeiro. Rev Saude Publica 45:31–39. 10.1590/S0034-89102011000100004 21181049

[19] Dye C, Scheele S, Dolin P, Pathania V, Raviglione MC. Consensus statement. Global burden of tuberculosis: estimated incidence, prevalence, and mortality by country. WHO Global Surveillance and Monitoring Project. JAMA. 18 de agosto de 1999;282(7):677–86. 10.1001/jama.282.7.677 10517722

[20] Ministério da Saúde [Internet]. Disponível em: https://www.gov.br/saude/acl_users/credentials_cookie_auth/require_login?came_from=https%3A//www.gov.br/saude/pt-br/centrais-de-conteudo/publicacoes/svsa/tuberculose/manual-de-recomendacoes-e-controle-da-tuberculose-no-brasil-2a-ed.pdf/view. Accessed 3 July 2026

[21] Esmaeilzadeh M, Dadak M, Atallah O, Möhn N, Skripuletz T, Hartmann C, et al. IgG4-related hypertrophic pachymeningitis with tumor-like intracranial and intracerebral lesions. Acta Neurochir (Wien). outubro de 2022;164(10):2781–7. 10.1007/s00701-022-05340-5 PMC9519706 35974231

[22] Wang Y, Yang Y, Guo X, You H, Xie M, Zhang S et al (2025) Clinical and prognostic profiles in immune-mediated hypertrophic meningitis: a retrospective analysis of 92 cases. Clin Rheumatol julho de 44(7):3073–3081. 10.1007/s10067-025-07531-8 40504382

[23] AbdelRazek MA, Venna N, Stone JH (2018) IgG4-related disease of the central and peripheral nervous systems. Lancet Neurol fevereiro de 17(2):183–192. 10.1016/S1474-4422(17)30471-4 29413316

[24] Wallace ZS, Deshpande V, Mattoo H, Mahajan VS, Kulikova M, Pillai S et al (2015) IgG4-related disease: clinical and laboratory features in one hundred twenty-five patients. Arthritis Rheumatol setembro de 67(9):2466–2475. 10.1002/art.39205 PMC4621270 25988916

[25] Wallace ZS, Miles G, Smolkina E, Petruski-Ivleva N, Madziva D, Cook C et al (2023) Incidence, prevalence and mortality of IgG4-related disease in the USA: a claims-based analysis of commercially insured adults. Ann Rheum Dis julho de 82(7):957–962. 10.1136/ard-2023-223950 37137671

[26] Lemos-Silva V, Araújo PB, Lopes C, Rufino R, da Costa CH (2011) Epidemiological characteristics of sarcoidosis patients in the city of Rio de Janeiro, Brazil. J Bras Pneumol Publicacao Of Soc Bras Pneumol E Tisilogia 37(4):438–445. 10.1590/s1806-37132011000400005 21881733

[27] Ramos-Casals M, Pérez-Alvarez R, Kostov B, Gómez-de-la-Torre R, Feijoo-Massó C, Chara-Cervantes J, et al. Clinical characterization and outcomes of 85 patients with neurosarcoidosis. Sci Rep. 2 de julho de 2021;11(1):13735. 10.1038/s41598-021-92967-6 PMC8253777 34215779

[28] Yokoseki A, Saji E, Arakawa M, Kosaka T, Hokari M, Toyoshima Y et al (2014) Hypertrophic pachymeningitis: significance of myeloperoxidase anti-neutrophil cytoplasmic antibody. Brain fevereiro de 137(2):520–536. 10.1093/brain/awt314 24271323

[29] Pearce FA, Craven A, Merkel PA, Luqmani RA, Watts RA. Global ethnic and geographic differences in the clinical presentations of anti-neutrophil cytoplasm antibody-associated vasculitis. Rheumatology. 1° de novembro de 2017;56(11):1962–9. 10.1093/rheumatology/kex293 28968886

[30] Redondo-Rodriguez R, Mena-Vázquez N, Cabezas-Lucena AM, Manrique-Arija S, Mucientes A, Fernández-Nebro A (2022) Systematic review and metaanalysis of worldwide incidence and prevalence of antineutrophil cytoplasmic antibody (ANCA) associated vasculitis. J Clin Med 11(9):2573. 10.3390/jcm11092573 PMC9106044 35566698

[31] Esclerose Múltipla — Ministério da Saúde [Internet]. Disponível em: https://www.gov.br/saude/pt-br/assuntos/pcdt/e/esclerose-multipla/view. Accessed 4 July 2026

